# The Development of Self-Identity (‘I’) in Children with Autism Spectrum Disorder: Clinical Implications for Self-Regulation

**DOI:** 10.1192/j.eurpsy.2026.10556

**Published:** 2026-07-31

**Authors:** J. M. Pałasz

**Affiliations:** Pareo Centrum Terapii, Kraków, Poland

## Abstract

**Introduction:**

The emergence of self-identity (‘I’) represents a fundamental milestone in child development. In neurotypical children, self-recognition and differentiation from others typically precede more advanced processes of emotional and behavioral self-regulation. However, in children with Autism Spectrum Disorder (ASD), the trajectory of self-identity development often follows an atypical path, influencing both communication and regulatory processes.

**Objectives:**

To examine how alterations in self-identity development in children with ASD affect their capacity for emotional and behavioral self-regulation, and to discuss therapeutic implications for clinical practice in child psychiatry.

**Methods:**

Clinical observations and therapeutic case studies of children aged 2–9 years with ASD were included in the analysis. The focus was on language acquisition, self-referential communication (use of “I”, “me”), and parental reports concerning self-awareness. Therapeutic interventions comprised structured speech and language therapy, parent-mediated strategies, and interdisciplinary collaboration with psychiatrists and psychologists.

**Results:**

Children who exhibited delayed or atypical self-identity development presented greater challenges in self-regulation, including difficulties in frustration tolerance, impulse control, and emotional expression. Conversely, interventions targeting early self-referential communication and identity development were associated with improved capacity for self-regulation, enhanced social reciprocity, and reduced behavioral crises.

**Conclusions:**

The development of self-identity is a key factor in the regulation of emotions and behavior in children with ASD. Integrating speech and language therapy focused on self-awareness into psychiatric care can support both communication and clinical outcomes. An interdisciplinary approach is essential to address the complex interplay between identity, communication, and regulation.

**Disclosure of Interest:**

None Declared

